# Nanostructured LiFe_5_O_8_ by a Biogenic Method for Applications from Electronics to Medicine

**DOI:** 10.3390/nano11010193

**Published:** 2021-01-14

**Authors:** Silvia Soreto Teixeira, Manuel P. F. Graça, José Lucas, Manuel Almeida Valente, Paula I. P. Soares, Maria Carmo Lança, Tânia Vieira, Jorge Carvalho Silva, João Paulo Borges, Luiza-Izabela Jinga, Gabriel Socol, Cristiane Mello Salgueiro, José Nunes, Luís C. Costa

**Affiliations:** 1 I3N and Physics Department, University of Aveiro, 3810-193 Aveiro, Portugal; silvia.soreto@ua.pt (S.S.T.); mpfg@ua.pt (M.P.F.G.); jmflucas7@gmail.com (J.L.); mav@ua.pt (M.A.V.); 2 CENIMAT/I3N, Departamento de Ciência dos Materiais, Faculdade de Ciências e Tecnologia, Universidade Nova de Lisboa, 2829-516 Caparica, Portugal; pi.soares@fct.unl.pt (P.I.P.S.); mcl@fct.unl.pt (M.C.L.); ts.vieira@fct.unl.pt (T.V.); jcs@fct.unl.pt (J.C.S.); jpb@fct.unl.pt (J.P.B.); 3 National Institute for Laser, Plasma and Radiation Physics, RO-077125 Magurele, Romania; izabela.jinga@inflpr.ro (L.-I.J.); gabriel.socol@inflpr.ro (G.S.); 4 Veterinary Sciences Institute, Ceará State University, Fortaleza 60714-903, Brazil; crismelloacp@gmail.com (C.M.S.); nunesuece@gmail.com (J.N.)

**Keywords:** lithium ferrite, proteic route, coconut water powder, dielectric spectroscopy, magnetic hyperthermia, specific absorption rate, cellular viability

## Abstract

The physical properties of the cubic and ferrimagnetic spinel ferrite LiFe_5_O_8_ has made it an attractive material for electronic and medical applications. In this work, LiFe_5_O_8_ nanosized crystallites were synthesized by a novel and eco-friendly sol-gel process, by using powder coconut water as a mediated reaction medium. The dried powders were heat-treated (HT) at temperatures between 400 and 1000 °C, and their structure, morphology, electrical and magnetic characteristics, cytotoxicity, and magnetic hyperthermia assays were performed. The heat treatment of the LiFe_5_O_8_ powder tunes the crystallite sizes between 50 nm and 200 nm. When increasing the temperature of the HT, secondary phases start to form. The dielectric analysis revealed, at 300 K and 10 kHz, an increase of $\varepsilon^{\prime }$ (≈10 up to ≈14) with a tan δ almost constant (≈0.3) with the increase of the HT temperature. The cytotoxicity results reveal, for concentrations below 2.5 mg/mL, that all samples have a non-cytotoxicity property. The sample heat-treated at 1000 °C, which revealed hysteresis and magnetic saturation of 73 emu g^−1^ at 300 K, showed a heating profile adequate for magnetic hyperthermia applications, showing the potential for biomedical applications.

## 1. Introduction

Lithium ferrite, formed in the cubic crystal system as LiFe_5_O_8_, is a very important and valuable material for numerous technologies and devices since it holds very attractive electric and magnetic properties. It belongs to the class of soft magnetic materials with high magnetization, high Curie temperature (620 °C), a square hysteresis loop, and low microwave dielectric losses [1,2] and has been leading the field of microwave applications [3,4]. Lithium ferrite is a low-cost and very attractive substitute for yttrium iron garnet (YIG) and other spinel ferrites and is also used in rod antennas, power transformers, and read/write heads for high-speed digital tapes due to its high resistivity, mechanical hardness, and chemical stability [5,6]. Other emerging applications of this material comprehend ferrofluid technology [7], gas sensors [8], contrast enhancement of magnetic resonance imaging (MRI), and magnetically guided drug delivery [9,10]. Bringing more into focus the electric properties of lithium ferrite, this is considered a very promising cathode material to be used in rechargeable Li-ion batteries, as it presents low dielectric losses [11,12,13].

Two crystalline forms occur in the spinel-type lithium ferrite. In the “ordered” form, the α-LiFe_5_O_8_ (space group P4_1_32/P4_3_32), has Fe^3+^ ions occupying octahedral 12d and tetrahedral 8c sites, while Li^+^ ions are only located at the octahedral 4b positions in the cubic primitive unit cell. The “disordered” β-LiFe_5_O_8_ has an inverse spinel structure (space group Fd3m), in which Fe^3+^ is located at tetrahedral 8a positions, and Li^+^ and Fe^3+^ are randomly distributed over the 16d octahedral sites [14]. 

Due to the high temperature (≥1200 °C) required for the production of lithium ferrite through the solid-state method, it is one of the most challenging ferrites to prepare, having in consideration the volatility of lithium above 1000 °C, which may lead to undesirable electric and magnetic properties [15,16]. Consequently, there is a great deal of interest in developing lower temperature preparation methods that can obtain good-quality LiFe_5_O_8_. Its synthesis has already been accomplished by the citrate precursor [14,17], sol-gel [18,19], autocombustion [20,21], and hydrothermal [13] methods, with the properties of the obtained LiFe_5_O_8_ depending to some degree on the employed preparation method.

Herein we report an ecologically friendly powdered coconut water (PCW)-mediated sol-gel route, for the preparation of LiFe_5_O_8_ with good quality and interesting properties.

Coconut water is the liquid contained in a coconut fruit. It is composed of water and both organic and inorganic compounds. The main fraction of soluble solids in coconut water is sugars [22,23], with sucrose, sorbitol, glucose, and fructose being the major ones present in mature coconut water [24,25]. Also present are minor sugars that include mannose, xylose, and galactose. The second major constituents of coconut water are minerals [26]. Coconut water is also composed of free or protein integrating amino acids (aa), with alanine, arginine, cysteine, and serine being present in higher concentrations [27,28]. These free aa have inherent properties and play an important role in conducting and assembling the magnetic nanoparticles (MNPs), acting as a surfactant, suspension stabilizer, and nucleation medium [29]. The growth factors (phytohormones) present in coconut water, gibberellins and auxins, mainly 3-indole acetic acid (IAA), together with maltodextrin added to the processing of obtaining powdered coconut water (ACP), lend their atoms carbon, together with inulin (very rich in glucose), which enter the pentose cycle and become ATP. This will be the energy source of anabolic reactions and aggregate to metal ions promoting saturation of the medium with carbon atoms forming materials with tertiary bonds. Later they turn into disulfide bridge bonds that will not disintegrate when the material is subjected to very high temperatures. Unlike what happens with egg albumin, in the ACP there is the amino acid cysteine which is rich in disulfide bridges. All of these reactions facilitate the synthesis of LiFe_5_O_8_. Two proteins are known to be present in coconut water, namely peroxidase and tyrosinase (polyphenoloxydase) [26], although other unknown proteins are also present. The total protein contents in dry samples are (i) young coconut water 2.19 g/100 g and (ii) mature coconut water 1.13 g/100 g [30]. The advantages of using coconut water as a sol-gel precursor for the production of ceramic materials are (i) high sugar concentration that promotes the gelation process, (ii) the presence of amino acids and proteins that can bind/complex metal ions, thus promoting the homogeneous distribution of the precursor ions, (iii) high abundance in nature, (iv) a renewable resource, (v) low cost, (vi) its availability on an industrial scale, and (vii) a simple production process of the powdered form. The main advantage of using powdered coconut water (PCW) instead of fresh or bottled coconut water for the sol-gel synthesis of ceramic materials is the control of the concentration of PCW in the process that ensures the reproducibility of the synthesis. This type of sol-gel route also shares the conveniences of the protein-type sol-gel [31,32] (coconut water, gelatin) or Pechini type [33] (citric acid with ethylene glycol) since these routes can increase the quality of the produced materials and reduce synthesis costs, temperature [34], and environmental contamination. These types of sol-gel routes are an alternative to the conventional sol-gel method in which metal alkoxide precursors are used. These precursors are toxic, have high synthesis costs, are not easy to obtain, and have a fast hydrolysis rate that makes it difficult to control the compositional homogeneity required during the material production process [35].

The coconut water-assisted sol-gel method, which is based on the principles of green chemistry, has been successfully used in the preparation of various ceramic materials. To name a few, the synthesis of SrFe_12_O_19_ [36], BaFe_12_O_19_ [37], and Eu-doped SrAl_2_O_4_ [38] has been achieved by using this method. Coconut water also finds application in nanotechnology, more particularly in the synthesis of nanoparticles (NPs). Gold [39], silver [40], BaTiO_3_ [41], Y_2_O_3_:Nd^3+^ [42], and superparamagnetic iron oxide [43] NPs have been synthesized using coconut water.

An emerging field of interest of magnetic nanoparticles is in cancer treatment by magnetic hyperthermia (MHT) [44,45,46]. This therapy is undergoing intense study because it is a minimally invasive technique that allows localized treatment by a temperature increment. MHT efficiency strongly depends on MNP physical properties, such as particle size and magnetic properties, which depend on the inductive external field frequency, translated by magnetic hysteresis losses that are crucial for the specific absorption rate (SAR), responsible for the local temperature increment.

One of the main purposes of this work was to investigate the viability of the PCW-mediated sol-gel route for the preparation of lithium ferrite. The influence of the calcination temperature employed in the pellets was evaluated by correlating the electric properties with its structural and morphological characteristics. The dielectric constant, dielectric loss, and conductivity dependence with temperature and frequency, in the case of the alternating field, were investigated for all samples, within the setup measurement range. This was done having in mind that this is a very attractive cathode material to be used in rechargeable Li-ion batteries. Also in this work, we expected to gain insight into the potentiality (fitness or application potential) to be applied in MHT. 

## 2. Materials and Methods

### 2.1. Ferrite Preparation

Lithium ferrite (LiFe_5_O_8_) powders were prepared by a sol-gel route known as the biogenic or proteic route, using iron (III) nitrate (Fe(NO_3_)3·9H_2_O) and lithium nitrate (LiNO_3_) (Merck KGaA, Darmstadt, Germany) as raw materials, weighted taking into account the stoichiometry of the reaction. First of all, the metal nitrates were dissolved in the powdered coconut water (PCW), (*Cocos nucifera* L.) solution with a concentration of 0.59 mol.dm^−3^, corresponding to the critical micellar concentration, and then were mixed using a magnetic stirrer in two steps: (i) T = 80 °C, ∆t= 2 h and (ii) T = 100 °C, ∆t =1 h, until the formation of a viscous brown gel. To dry, removing all solvent from the as-synthesized powders, a heat-treatment process at 350 °C, for 1 h, was performed. The dried powders were pressed, forming disks with a diameter of 10 mm and a thickness of about 2 mm. Those disks and powders were jointly submitted to different heat treatments, changing the maximum temperature between 400 and 1000 °C, using a constant dwell time of 4 h and a heating rate of 5 °C/min, under atmospheric pressure. The cooling process was performed in agreement with the furnace thermal inertia characteristic when it is switched off. 

Thermal measurements were carried out by *Hitachi STA7300* equipment, in nitrogen atmosphere, in a range of temperatures from room temperature up to 1250 °C, with an heating rate of 5 °C/min.

### 2.2. Structural and Morphological Characterization

The disks and powders were submitted to a structural characterization with different techniques: X-ray diffraction (XRD), using an X’Pert MPD Philips diffractometer (CuKα radiation, λ = 1.54060 Å) and with the X’Pert HighScore PANalytical software to perform the Rietveld refinement. X-ray photoelectron spectroscopy (XPS) was performed with ESCALAB Xi+ (Thermo Scientific Surface Analysis) equipped with a multichannel hemispherical electron analyzer (dual X-ray source) working with Al Kα radiation (hν = 1486.2 eV) and using C 1s (284.4 eV) as energy reference. XPS data were recorded on slightly pressed powders that had been outgassed in the pre-chamber of the setup at room temperature at a pressure of <2 × 10^−8^ Torr to remove the chemisorbed water from their surfaces. The surface chemical compositions and oxidation states were estimated from the XPS spectra by calculating the integral of each peak after subtraction of the “S-shaped” Shirley-type background using the appropriate experimental sensitivity factors using Avantage software (version 5.978). Micro-Raman spectroscopy, using a Jobin–Yvon spectrometer, was performed at room temperature in backscattering geometry, with the microscope objective (50×) focusing the exciting light (λ = 532 nm) onto the sample (spot diameter <0.8 µm). Plasma lines were removed by a filter. 

The free surface morphology of the disks was analyzed by scanning electron microscopy (SEM), using a Vega 3 TESCAN microscope. Previous to the microscopic observation, all samples were coated with carbon to promote surface conductivity.

### 2.3. Electrical Characterization

Impedance spectroscopy was performed using an Agilent 4294A precision impedance analyzer, in the C_p_-R_p_ configuration, in the frequency range of 100 Hz–1 MHz and temperature from 200 to 360 K, using a bath-cryostat in which the samples are in a helium atmosphere to improve the heat transfer and avoid moisture. The samples in the disk form were used, where the opposite surfaces were painted with conductive silver paste, forming the electrodes.

### 2.4. Magnetic Characterization

Magnetic hyperthermia measurements were obtained using a DM100 series from the nB nanoScale Biomagnetics apparatus. The heating ability of LiFe_5_O_8_ samples prepared with different HT using concentrations ranging from 0.625 to 10 mg/mL was measured using an alternating current (AC) magnetic field of 24 kA.m^−1^, with a frequency of 418.5 kHz for 10 min. Each sample was immersed in 1 mL of ultrapure water and ultra-sonicated before each measurement. Magnetic measurements were made using a vibrating sample magnetometer (VSM). The isothermal magnetization curves were obtained for magnetic fields (H) up to 10 T at 300 K.

### 2.5. Cytotoxicity Analysis

To evaluate the cytotoxicity of the LiFe_5_O_8_ samples, the assays were performed according to standard ISO-10993 “Biological evaluation of medical devices—Part 5: Tests for in vitro cytotoxicity”. The assays were performed using the extract method and Vero cells (monkey renal epithelial cells). To produce the extract, an initial concentration of 10 mg/mL of each sample was used. Each sample was placed in 1 mL of Dulbecco’s modified Eagle’s medium (DMEM, Sigma Aldrich) at 37 °C for 48 h. Cells were seeded at a density of 20,000 cell cm^−2^ in 96-well plates and grown in DMEM supplemented with 10% fetal bovine serum, 1% Penicillin-Streptomycin, sodium pyruvate (100 mM, Life Technologies), and GlutaMAX™ Supplement (Life Technologies) followed by incubation at 37 °C in 5% CO_2_ for 24 h. Four dilutions of the extract were used: 5, 2.5, 1.25, and 0.625 mg mL^−1^. For each concentration, 5 replicas were performed. After this period, the medium was removed and a resazurin solution containing 90% of complete culture medium, as described above, and 10% of a 0.2 mg mL^−1^ resazurin solution in PBS was added to each well. After 2 h incubation, the absorbance was measured at 570 and 600 nm. Negative control cells were incubated with a complete medium. Positive control cells were treated with 10% DMSO to cause cell death. Cell viability is expressed as a percentage of the negative control, given by [% cell viability = treated cells/control cells × 100].

## 3. Results and Discussion

### 3.1. Thermal and Structural Analysis

**Figure 1:**
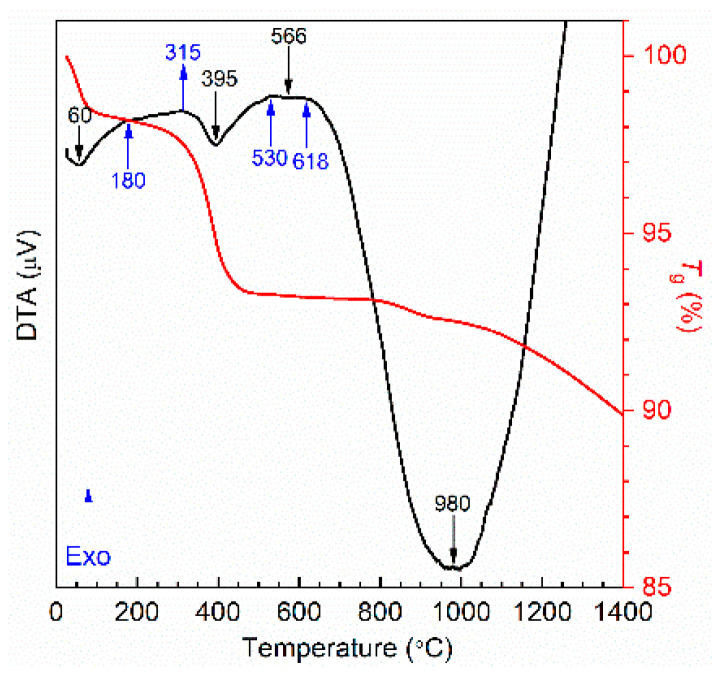
DTA/TGA thermograms of the obtained powders.

Figure 1 shows the result of the thermal analysis of the green powder. In these DTA/TGA thermograms, the exothermic bands, which are not associated with weight loss and are centered around 315 and 515–618 °C, suggest the formation of crystalline structures. Until 1400 °C, there is a total weight loss of 10%, approximately, in three steps: first, around 60 °C, which should be related to water evaporation, the second nearby 395 °C, which is related to the decomposition of the organic matter, and the third at 980 °C, which could be related to a crystal phase transformation. Based on these results, the selected temperatures for the heat treatments were 400, 600, 800, and 1000 °C.

**Figure 2:**
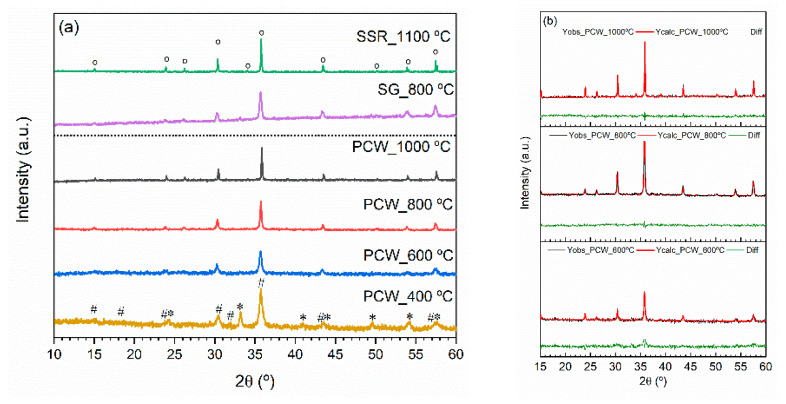
(**a**) XRD diffractograms of the samples heat-treated between 400 and 1000 °C and of the samples prepared by sol-gel (SG_800 °C) and by solid-state reaction (SSR_1100 °C) [48] (o LiFe_5_O_8_; * Fe_2_O_3_; # Fe_2_._67_O_4_); (**b**) Rietveld refinement of the samples treated at 600, 800 and 1000 °C, showing the experimental data, Y_obs_, the refinement adjust, Y_calc_, and the difference between them, Diff.

The XRD diffractograms of the heat-treatment samples, shown in Figure 2a, present the diffraction peaks characteristic of the disordered lithium ferrite crystalline phase (β-LiFe_5_O_8_) in the samples heat-treated at 600, 800, and 1000 °C [47]. The sample heat-treated at 400 °C shows the diffraction peaks characteristic of iron oxides crystalline phases, Fe_2_O_3_ and Fe_2_._67_O_4_. As in this sample, the lithium ferrite crystalline phase was not detected, and all the following analyses were performed in the samples treated at temperatures above 400 °C. Figure 2a also shows, for comparison analysis, the results of previous works where the LiFe_5_O_8_ was prepared by Pechini [48] and by solid-state reaction [15]. As can be seen, the diffraction peaks agree with the previous results once only the disordered phase was detected.

Rietveld refinements were performed in the XRD diffractograms to estimate the lattice parameters, the crystallite sizes, by both Scherrer ($L_{Shc}$) and Williamson–Hall (WLH) methods and the micro-strain (e), related to crystal imperfections and distortion. The obtained results are summarized in Table 1. The average crystallite sizes of lithium ferrite crystal phase determined by the Scherrer method, $L_{Shc}$, uses the following Debye–Scherrer equation [49]:

(1) $$L_{Sch}=\frac{N\lambda }{\beta cos\;\theta }$$

where λ is the wavelength of X-ray radiation (1.54184 Å), N is a numerical factor frequently referred to as the crystallite-shape factor [49], where *n* = 0.9 is a good approximation for particles with a spherical habit [50], θ is the diffraction angle, and *β* is the full width half maximum (FWHM) of the diffracted peaks, corrected by the relation $\beta =\sqrt{W_{exp}^{2}-W_{inst}^{2}}$, where $W_{exp}^{2}$ and $W_{inst}^{2}$ are the experimental and the instrumental width. The instrumental width was determined using the LaB_6_ powder standard pattern (SRM 660—National Institute of Standard Technology). According to Figure 2b and the parameters in Table 1, the quality of the refinements was good once $R_{exp}\leq R_{p}$ and the goodness of the fit, $\chi^{2}={\left(\frac{R_{wp}}{R_{exp}}\right)}^{2}$ is higher and closer to 1 [51].

As thermodynamically expected, the LiFe_5_O_8_ crystallite sizes increase with the increase of the heat-treatment temperature. Nevertheless, the PCW_1000 °C sample presents a crystallite size higher than 100 nm, indicating that the Debye–Scherrer method is not appropriate to calculate this characteristic, as the experimental FWHM of the diffraction peaks is out of the limitation of the instrumental ones provided by the standard pattern LaB_6_. The Williamson–Hall method, for determination of the β parameter, has the advantage to the Debye–Scherrer method of taking into account the strain contribution to the X-ray line broadening [52], i.e., $\beta_{struc}=\beta_{size}+\beta_{strain}$, where $\beta_{size}$ is obtained from the Debye–Scherrer equation (Equation (1)) and $\beta_{strain}=4etan\;\theta$, e being a coefficient related to the crystallite strain effect. Therefore, it is possible to determine the crystallite size ($L_{WH}$) and the lattice microstrain by the slope of “β.cosθ“ in function of “4.sin θ” [53] and the interception of the linearization line at the origin, according to Equation (2):

(2) $$\beta_{struc}cos\;\theta =\frac{N\lambda }{L_{Sch}}+4esin\;\theta$$

The crystallite sizes, determined by the Williamson–Hall method, for the samples heat-treated at 600 and 800 °C, are smaller than 100 nm, being smaller for the sample heat-treated at 800 °C. In addition, in this sample, the strain value (slope Equation (2)) is negative, meaning that it presents a compressive strain, while if the value is positive indicates the presence of a tensile strain (samples PCW_600 °C and PCW_1000 °C) [53].

**Figure 3:**
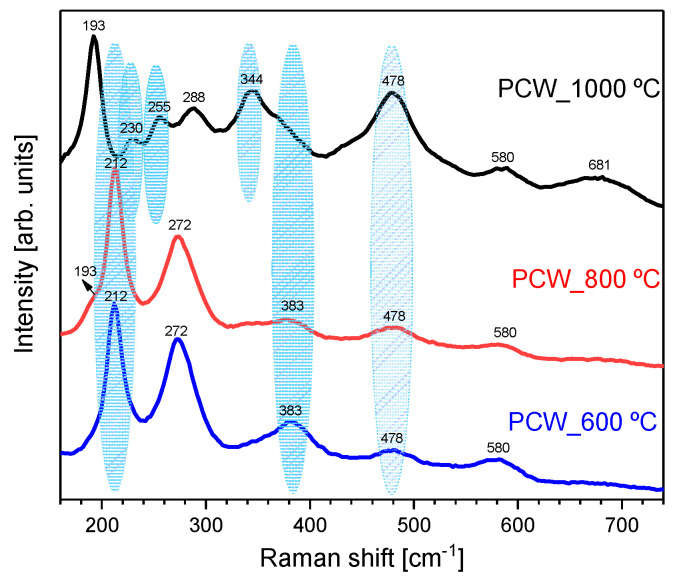
Raman spectroscopy of samples heat-treated at 600, 800, and 1000 °C.

XRD results are corroborated by the Raman results (Figure 3), where the structural vibrations related to lithium ferrite were detected. Moreover, the Raman spectra present additional structural information by revealing the presence of both ordered and disordered LiFe_5_O_8_ crystal phases. The vibration bands, highlighted in blue color, are only characteristics of the α-LiFe_5_O_8_ crystalline phase and the others characteristic of the disordered crystalline phase [54]. The sample heat-treated at 1000 °C shows a more expression of the α-LiFe_5_O_8_ crystalline phase.

**Figure 4:**
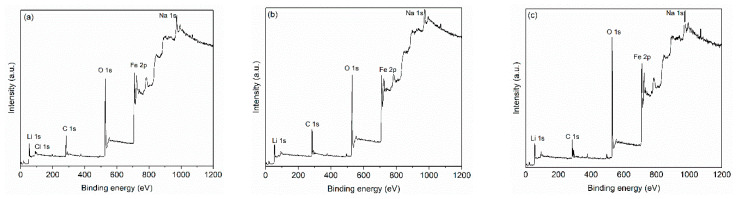
XPS spectra of the samples heat-treated at (**a**) 600 °C, (**b**) 800 °C, and (**c**) 1000 °C.

**Figure 5:**
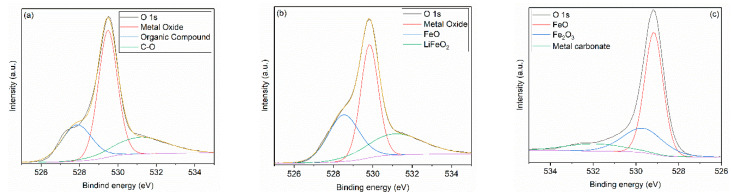
XPS O 1s high-resolution spectra of the samples heat-treated at (**a**) 600 °C, (**b**) 800 °C, and (**c**) 1000 °C.

Figure 4 shows the XPS survey spectra, revealing the presence of Li, Fe, C, O, Cl, and Na elements, in all samples. For the sample heat-treated at 600 °C, the deconvolution of high-resolution spectra of oxygen (Figure 5a), recorded between 531.5–532 eV, shows the presence of some impurities, such as metal oxides, and traces of organic compounds because of residues from the synthesis route of the lithium ferrite. In the case of the samples treated at higher temperatures (800 °C and 1000 °C), the characteristics’ peaks related to impurities disappear, and the structure of iron oxides is distinguished by its specific transition in O 1s high-resolution spectra at 529.8 eV and 530.3 eV for FeO and Fe_2_O_3_, respectively. However, the presence of metal carbonates was identified since the decomposing temperature of these compounds can be around 1200 °C [55,56].

High-resolution spectra of lithium (Figure S1) unveiled the peaks for Li metal at 54.8 eV, oxide (Li_2_O) at 55.6 eV, and carbonate (Li_2_CO_3_) at 55.2 eV [56].

Lithium ferrites are CO_2_ captors with different behaviors, depending on their structure. For example, the Li_5_FeO_4_ phase is able to chemisorb large amounts of CO_2_ at temperatures between 300 and 800 °C [57]. The reactions that occur are:

2LiFeO_2_ + CO_2_ → Li_2_CO_3_ + Fe_2_O_3_ (3)

Li_5_FeO_4_ + 2CO_2_ → 2Li_2_CO_3_ + LiFeO_2_ (4)

Therefore, in the high-resolution spectra of the samples treated at 600 and 800 °C, lithium carbonate could be identified. At higher temperatures, over 900 °C, Li_2_CO_3_ decomposes into Li_2_O and CO_2_, and the Li_2_CO_3_ peak disappears [55].

The high-resolution spectra of Fe 2p reveal a spinel structure with a typical peak at 717.4 eV. The peaks for Fe^3+^ at 710.8 eV corresponding to 2p3/2 transition and Fe^2+^ at 709.3 eV and 720 eV associated with 2p3/2 and 2p1/2 transitions could be identified in all iron spectra [55,56,57,58]. Additionally, since the lithium ferrite is prone to react with the chemosorbed CO_2_, the intensity of Fe^3+^ and Fe^2+^ peaks is more intense [55,56].

**Figure 6:**
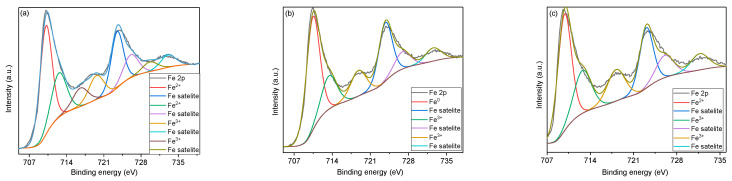
XPS Fe 2p high-resolution spectra of the samples heat-treated at (**a**) 600 °C, (**b**) 800 °C, and (**c**) 1000 °C.

In the high-resolution spectra of C 1s, the peaks corresponding to C-C at 284.8 eV and C-O-C at 286 eV are identified for all investigated samples (Figure 6 and Figure S2). The presence of organic impurities from the coconut water precursor is unveiled in the high-resolution spectra of C 1s for the samples thermally treated at 600 and 800 °C, a fact revealed also in the high-resolution spectra of O 1s (Figure 5). Due to the reaction of lithium ferrite with the chemosorbed CO_2_, Li_2_CO_3_ (289.6 eV) is observed in the high-resolution spectra of C 1s. Moreover, in the case of the sample treated at 1000 °C, metal carbonate is also identified in the high-resolution spectra of C 1s. The metal carbonate band is between 288–290 eV, and it can be from K_2_CO_3_, Na_2_CO_3_, and traces of Li_2_CO_3_. [55,56]. These results are in line with the analysis of the surface by the SEM-EDS technique, where potassium was detected in a vestigial quantity. It should be noticed that XPS is a surface characterization, which allows the determination of the abundance of chemical elements in the sample surface region (10 nm depth), their oxidation states, and bound characteristics. Due to the interaction with the environment, the surface of the lithium ferrite changes its composition by a chemical reaction made by the chemosorption of carbon dioxide obtained during the heat treatment.

### 3.2. Morphological Analysis

**Figure 7:**
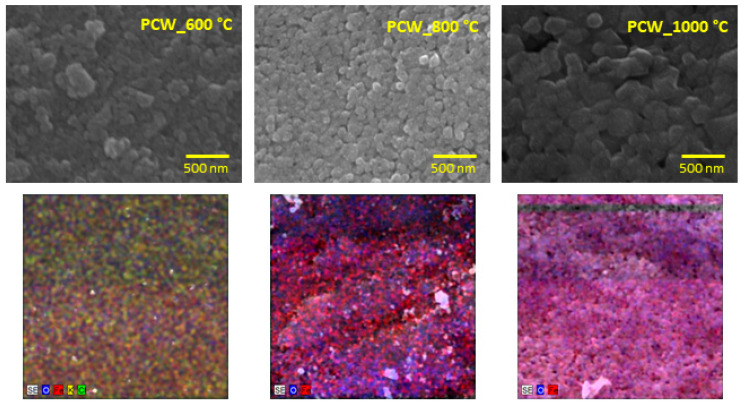
Surface morphology, by SEM, of the samples heat-treated at 600, 800, and 1000 °C, where the grains reach the nanoparticles’ size (**upper figures**); EDS mapping detects oxygen (**blue color**) and iron (**red color**) elements for all samples; potassium (**yellow color**) and chlorine (**green color**) are detected just for PCW_600 °C (**bottom figures**).

Figure 7 (upper micrographs) shows the SEM micrographs, where it is visible that the particle size increases with the temperature of heat treatment. All those samples have a prismatic grain habit, which is in accordance with the literature [48]. For the samples treated at 600 and 800 °C, the average size of the grains is around 80 nm, increasing to around 170 nm for the sample PCW_1000 °C. These results are in line with the crystalline sizes calculated from the XRD patterns. 

In Figure 7 (bottom micrographs), the elementary analysis is shown, automatically detected by EDS, where the carbon element was excluded once the samples were coated with it. From the SEM-EDS analysis, for all samples, the elements oxygen (from 61.37%_at_, sample PCW_600 °C, to 69.01%_at_, sample PCW_800 °C) and iron (from 30.99%_at_, sample PCW_600 °C, to 38.31%_at_, sample PCW_800 °C) were detected. Besides that, just for the sample PCW_600 °C, some vestigial elements such as potassium (0.20%_at_) and chlorine (0.12%_at_) were detected. This evidence is certainly related to the lithium ferrite synthesis route, once the powder of coconut water has a very rich composition in carbohydrates, amino acids, vitamins, proteins, and minerals, potassium being one of the major mineral constituents. In the mineral composition, potassium is the major constituent [59]. Given that EDS just detects elements with an atomic number higher than 5, only the automatic search was made including the elements with an atomic number higher than that.

### 3.3. Dielectric Analysis

**Figure 8:**
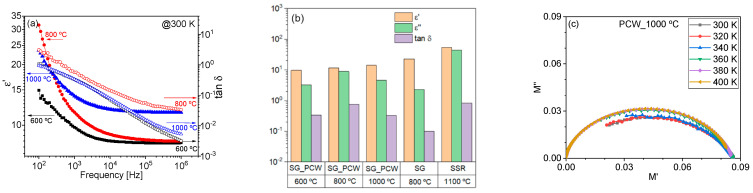
(**a**) Dielectric constant (*ε*’) and dielectric loss (*tan δ*) for all samples; (**b**) Comparison of LiFe_5_O_8_ synthesis by different methods: sol-gel (proteic (SG_PCW) and Pechini (SG) routes) and solid-state reaction (SSR); (**c**) Nyquist plot of the sample heat-treated at 1000 °C, at temperatures from 300 to 400 K.

Impedance spectroscopy studies were used to determine the real, $\varepsilon^{\prime }$, and the imaginary, $\varepsilon^{\prime \prime }$, parts of the complex permittivity, $\varepsilon^{*}$. These physical characteristics were achieved using the expression (5), taking the measured values of the C_p_ and R_p_ [60]:

(5) $$\varepsilon^{*}=\varepsilon^{\prime }+j\varepsilon^{\prime \prime }=\frac{d}{A}\frac{C_{p}}{\varepsilon_{0}}+j\frac{d}{A}\frac{1}{{\omega R}_{p}\varepsilon_{0}}$$

where $\varepsilon_{0}$ is the empty space permittivity, *d* the sample thickness, A is the electrode area, and ω the angular frequency, respectively. Figure 8 shows the obtained values, at room temperature, for $\varepsilon^{\prime }$ and $\tan\;\delta =\varepsilon^{\prime \prime }/\varepsilon^{\prime }$. 

For electronic applications, namely for energy storage and distribution systems, the best balance between the dielectric constant and the loss tangent must be achieved. In the present case, the sample PCW_1000 °C is the promising one, since it has the highest dielectric constant for frequencies above 1 kHz and low dielectric losses. It is suggested that this behavior is related to the presence of the LiFe_5_O_8_ disorder phase [48]. Comparing with the solid-state reaction the lithium ferrite crystalline phase is obtained at the lowest temperature of heat treatment for the proteic route, which is an advantage. Furthermore, the particles obtained by this proteic route have a smaller size, at the nanoscale, revealing that this eco-friendly method is suitable for producing nanosize particles that can be used in electronic applications. It is also important to mention that, in all samples, a relaxation process was visible, as shown in the Nyquist plot of the sample PCW_1000 °C (Figure 8c). This relaxation process, thermally activated, is defined in the frequency window, for temperatures above 360 K presenting a typical Cole–Cole behavior as the semicircle center is dislocated below the “x” axis [61].

### 3.4. Biological Analysis

**Figure 9:**
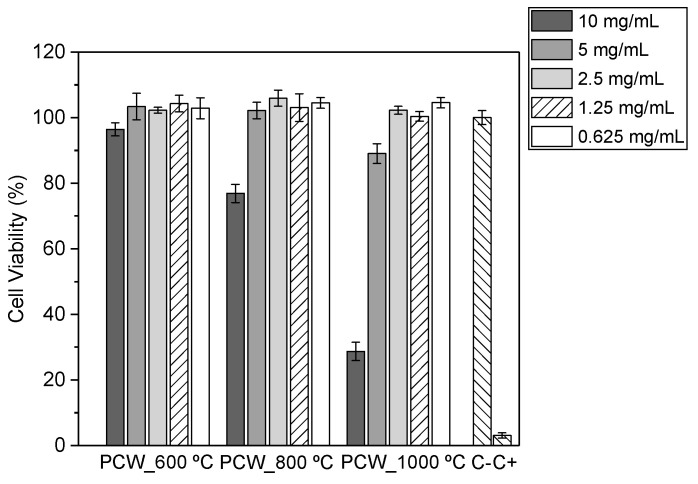
Cell viability in the function of powder heat treatment.

An analysis of the cytotoxicity of the prepared samples was performed, revealing, for concentrations below 2.5 mg/mL, that all samples have a non-cytotoxicity behavior (Figure 9). This should be related to the formation and relative quantity of lithium ferrite in the ceramic matrix, indicating that the presence of such crystallites, even in nanosized scale, affects negatively the cytotoxicity characteristics. 

### 3.5. Magnetic Analysis

**Figure 10:**
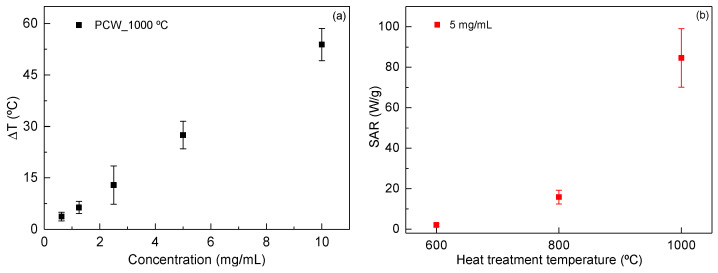
(**a**) Temperature increase generated by LiFe_5_O_8_ ferrite after heat treatment at 1000 °C. (**b**) Specific absorption rate of LiFe_5_O_8_ ferrites after heat treatment at different temperatures from 600 °C up to 1000 °C using a concentration of 5 mg/mL in ultrapure water.

Magnetic hyperthermia assays were performed to evaluate the heating ability of LiFe_5_O_8_ powders treated at different temperatures and their potential to be used as magnetic hyperthermia agents. In Figure 10a, the heating performance of LiFe_5_O_8_ ferrite after heat treatment at 1000 °C is shown. Different concentrations were tested, demonstrating a correlation between ferrite concentration and generated temperature. This result is agrees with previous studies using iron oxide nanoparticles as magnetic hyperthermia agents [46,62,63,64]. Figure 10b shows the relationship between the specific absorption rate (SAR) with the heat treatment temperature of lithium ferrites. Through SAR values, it is possible to evaluate the heating efficiency of a magnetic material following energy absorption during its exposure to an alternating magnetic field. The value is defined as the quantity of power absorbed by the sample per mass unit (W/g) and was calculated using the following equation:

(6) $$SAR\;\left(W/g\right)=\frac{C_{NP}m_{Fe}+C_{l}m_{l}}{m_{Fe}}{\left(\frac{dT}{dt}\right)}_{max}$$

where (dT/dt)max is the maximum gradient of the heating curve (obtained at adiabatic conditions), $C_{NP}$ is the specific heat of the magnetic material, $C_{l}$ is the specific heat of the liquid, $m_{l}$ is the fluid mass, and $m_{Fe}$ is the iron mass in the ferrite solution. 

**Figure 11:**
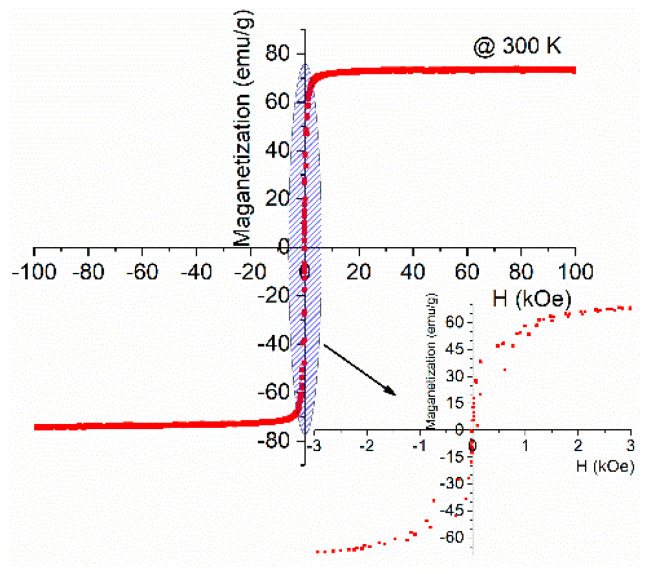
Hysteresis loop for sample PCW_1000 °C.

The results demonstrate that lithium ferrites heat-treated at 600 °C do not exhibit a heating profile adequate for magnetic hyperthermia applications since their SAR value is very low (2.0 W/g). The two highest temperatures of heat treatment (800 and 1000 °C) exhibit an increase in the heating ability, with a significant difference for ferrites treated at 1000 °C, with SAR about 80 W/g, which is similar to that obtained for Fe_3_O_4_ [45] and higher than those obtained for Fe_2_O_3_ in a paper from Gu et al. [65]. This difference can be related to the fact that this sample exhibits a high grain size. The heat performance of the magnetic nanoparticles can be caused by (i) hysteresis, (ii) Neel relaxation or Brownian relaxation, or (iii) Eddy current losses by friction and viscous suspensions [66]. Figure 11 shows the hysteresis loop, obtained for the sample HT at 1000 °C, showing a magnetic saturation ($M_{S}$) of around 73 emu g^−1^ at 300 K and for H = 30 kOe with a coercivity (*H_C_*) of 0.06 kOe. It is important to note that the hysteresis loss occurs due to the irreversible magnetization process in the magnetic field, originated in particles with multiple magnetic domains [66]. These results are in agreement with previous studies [67]. The studied samples have a superparamagnetic behavior and do not have many magnetic domains. The samples under study have a small coercivity, with a hysteresis cycle also small, and a high saturation magnetization. This leads us to admit that the main source for heating the samples is the induced current (eddy currents). The induced voltage increases with the increase of the particle size, and this associated with the decrease of the resistance of the particle and the decreasing of the grain boundaries, with a higher resistance, causes an increase in energy dissipation (Joule effect). The value of the saturation magnetization value (Figure 11) is reached with low applied external fields (<5 Koe), which allows one to obtain a large f.e.m. with a low applied external field.

## 4. Conclusions

It was possible to synthesize lithium ferrite crystal phase by a novel eco-friendly and sustainable route, using a powder of coconut water. The prepared nanoparticles have high heat efficiency, especially the sample heat-treated at 1000 °C, showing the potential to be used in the treatment of cancer, by magnetic hyperthermia therapy. The heat performance of these magnetic nanoparticles can be attributed to Eddy current losses. Besides that, the cytotoxicity assay showed a non-toxicity behavior being a key advantage to be applied in this technique. 

Moving on to the electronic applications, the sample PCW_1000 °C is the promising one to store energy, since it has the highest dielectric constant and low dielectric losses, for frequencies above 1 kHz and 300 K.

## Figures and Tables

**Table 1:** Parameters of the Rietveld refinement, using Scherrer and Williamson–Hall methods.

Sample	*R_p_*	*R_wp_*	*R_exp_*	*a = b = c* (Å)	*α = β = γ* (°)	$\chi^{2}$	$L_{Sch}\;(\mathrm{nm})$	$L_{WH}\;(\mathrm{nm})$	$e_{WH}\;(\%)$
PCW_600 °C	3.073	4.044	3.657	8.33785 ± 0.00003	90	1.223	43.68	77.12	0.1733
PCW_800 °C	1.818	2.264	2.190	8.33450 ± 0.11445	90	1.069	56.54	40.63	0.0562
PCW_1000 °C	2.823	3.649	3.614	8.33321 ± 0.00003	90	1.019	237.40	146.65	0.0192

## Supplementary Materials

The following are available online at https://www.mdpi.com/2079-4991/11/1/193/s1, Figure S1. XPS Li 1s high resolution spectra of the samples HT at (a) 600 °C, (b) 800 °C and (c) 1000 °C; Figure S2. XPS C 1s high resolution spectra of the samples heat treated at (a) 600 °C, (b) 800 °C and (c) 1000 °C.
