# Supplementary material for: Nanostructured LiFe5O8 by a Biogenic Method for Applications from Electronics to Medicine
Source: Nanomaterials (Basel). 2021 Jan 14;11(1):193. doi: 10.3390/nano11010193 (PMC7828716; doi:10.3390/nano11010193)
Supplement: Supplementary file 1 [file nanomaterials-11-00193-s001.pdf]

nanomaterials

MDPI

Supplementary Materials: Nanostructured $LiFe_5O_8$ by a Biogenic Method for Applications from Electronics to Medicine

Silvia S. Teixeira [1], Manuel P. F. Graça [1], José Lucas [1], Manuel Almeida Valente [1], Paula I. P. Soares [2], Maria Carmo Lança [2], Tânia Vieira [2], Jorge Carvalho Silva [2], João Paulo Borges [2], Luiza-Izabela Jinga [3], Gabriel Socol [3], Cristiane Mello Salgueiro [4], José Nunes [4] and Luís C. Costa [1,*]

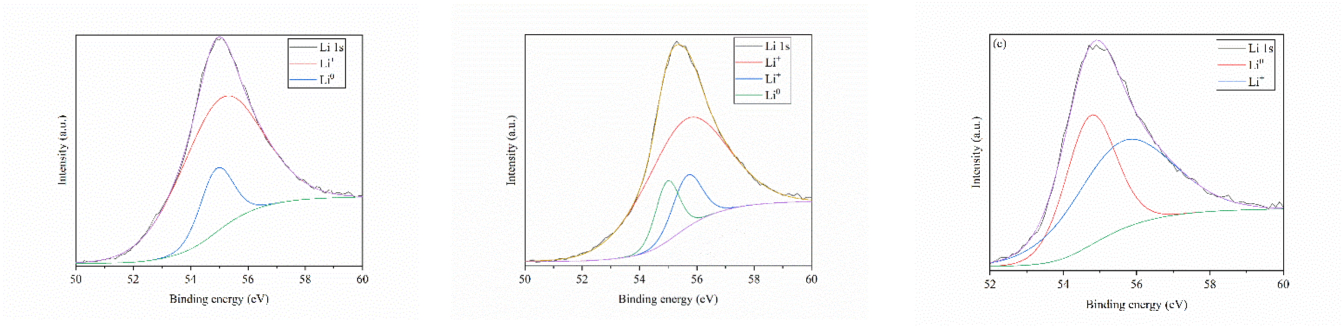

Figure S1. XPS Li 1s high resolution spectra of the samples HT at (**a**) 600 °C, (**b**) 800 °C and (**c**) 1000 °C.

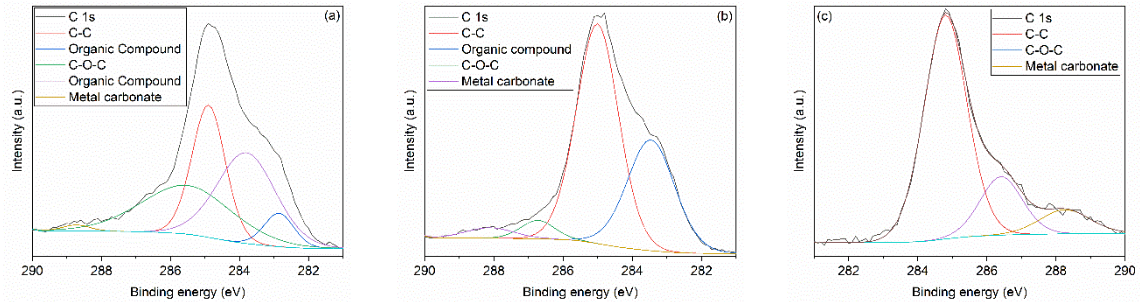

Figure S2. XPS C 1s high resolution spectra of the samples heat treated at (**a**) 600 °C, (**b**) 800 °C and (**c**) 1000 °C.
